# Maternal Compliance to Recommended Iron and Folic Acid Supplementation in Pregnancy, Sri Lanka: A Hospital-Based Cross-Sectional Study

**DOI:** 10.3390/nu12113266

**Published:** 2020-10-25

**Authors:** Malshani L. Pathirathna, Kuruppu M. S. Wimalasiri, Kayako Sekijima, Mieko Sadakata

**Affiliations:** 1 Department of Nursing, Faculty of Allied Health Sciences, University of Peradeniya, Peradeniya 20400, Sri Lanka; 2 Department of Food Science and Technology, Faculty of Agriculture, University of Peradeniya, Peradeniya 20400, Sri Lanka; swarnaw@pdn.ac.lk; 3 Department of Nursing, School of Health Sciences, Niigata University, 2-746 Asahimachi-dori, Chuo-ku, Niigata 951-8518, Japan; ksekijima@clg.niigata-u.ac.jp (K.S.); sadakata@clg.niigata-u.ac.jp (M.S.)

**Keywords:** anaemia, iron, folic acid, maternal compliance, Sri Lanka

## Abstract

Iron deficiency anaemia during pregnancy is a common public health problem that negatively affects maternal and newborn health. This study aims to identify the rate of maternal compliance with the recommended iron and folic acid (IFA) supplementation during pregnancy and to identify factors associated with maternal compliance and non-compliance. A hospital-based cross-sectional study was conducted among 703 women at 0–4 days postpartum. The prevalence of anaemia at the initial antenatal clinic (ANC) visit and at the third trimester were 20.8% and 44.9%, respectively. The rate of IFA supplementation compliance during pregnancy was 80.1%. Forgetfulness (66.9%) was the major reason for non-compliance, followed by side effects (15.7%). Maternal employment [OR (95%CI): 1.7 (1.00–2.89)], history of a low birth weight infant [OR (95%CI): 0.4 (0.19–0.9)] and history of anaemia [OR (95%CI): 0.4 (0.12–0.98] were significantly associated with maternal compliance with IFA supplementation. Only 26.6% of the participants adhered to dietary recommendations during the period when IFA supplements were provided. The rate of maternal compliance with IFA supplementation was high. However, the prevalence of maternal anaemia during pregnancy was also high, which was presumably due to poor dietary compliance despite high IFA supplementation compliance.

## 1. Introduction

Anaemia is one of the most common micro-nutrient deficiencies among pregnant women worldwide [1]. According to the World Health Organization (WHO) report on the worldwide prevalence of anaemia 1993–2005 (the latest data available to date), the global prevalence of anaemia for the general population is 24.8% (1620 million people) [2]. Southeast Asia reported the highest prevalence of anaemia in pregnant women (48.2%), while Africa reported the second highest prevalence (46.16%) in 2016 [3]. In Sri Lanka, the estimated prevalence of anaemia in pregnant women is 35.36% [3], which corresponds to a moderate prevalence according to the WHO [4,5]. Although anaemia is a multifactorial condition, iron deficiency is the most common cause. During pregnancy, a high load of iron is required to support the growing foetus and placenta, as well as to increase the maternal red blood cell mass. Consequently, pregnant women are at a considerable risk of developing iron deficiency anaemia, especially if their diets contain a low bioavailability of iron.

Anaemia during pregnancy increases the risk of low birth weight (LBW) infants, lowers the infant’s resistance to infection and can lead to poor cognitive development [6]. Iron supplementation, fortification of staple foods with iron, health and nutrition education, control of parasitic infections and improved sanitation are key interventions that can prevent iron deficiency anaemia during pregnancy [7]. A study carried out in Sri Lankan plantation sector reported increased beneficial effects of iron supplementation on haemoglobin concentration when women were provided with anthelminthic therapy during pregnancy [8]. However, many industrialised countries (e.g., the UK) have opposed universal iron supplementation in their national healthcare guidelines [9], because of the low prevalence of anaemia in these countries achieved by means of universal iron fortification programmes [10]. Although it is clear that the best approach to meet the increased iron demand during pregnancy is good nutrition that includes iron-rich foods, this is not always possible for low- and low- to middle-income populations in developing countries. Furthermore, the diets of most people in many South Asian countries, including Sri Lanka, rely heavily on cereals and legumes, both of which contain low amounts of bioavailable iron. As a result, the risk of iron deficiency anaemia in these countries is high. Therefore, the WHO recommends that all pregnant women in areas with a high prevalence of malnutrition receive 30–60 mg of elemental iron, together with 400 µg folic acid, beginning as early as possible during pregnancy and continuing until the birth of the child [11,12]. Iron supplementation should be accompanied by advice regarding appropriate dietary measures [12]. Consistent with the WHO’s recommendations, maternal healthcare guidelines in Sri Lanka recommend that all pregnant women receive daily supplements of 60 mg of elementary iron and 400 µg of folic acid, together with vitamin C, from the beginning of the 13th gestational week and continuing for 6 months after delivery [13]. An additional recommendation is that all pregnant women receive dietary advice regarding iron-rich foods, including animal and non-animal sources of dietary iron; the importance of consuming dietary iron together with citrus fruit or juice; and the avoidance of concomitant tea, coffee, or milk consumption with iron, as this inhibits iron absorption [14].

By strengthening and supporting policy measures aimed at maternal anaemia prevention, the totally free healthcare system in Sri Lanka provides all the necessary supplements (including IFA supplements, vitamin C tablets and calcium lactate supplements) through community ANCs for all pregnant women from the second trimester of pregnancy until 6 months postpartum. Although this universal supplementation programme has been in existence in Sri Lanka for several decades, the prevalence of maternal anaemia remains substantial (32.41% to 35.36% from 2006 to 2016) [3]. Therefore, the aim of this study is to identify factors related to maternal compliance with IFA supplementation and the underlying reasons for the poor efficacy of the IFA programme in Sri Lanka. The results should lead to improvements in the programme, and may be instructive for other countries in the region that are attempting to prevent maternal anaemia.

## 2. Materials and Methods

### 2.1. Design, Setting and Participants

This descriptive, hospital-based cross-sectional study was conducted in Kurungegala Provincial General Hospital, Sri Lanka between September 2018 and October 2019. Women who had given birth 0–4 days previously and were hospitalised in the postnatal ward following delivery of a singleton neonate were included in the study. Women with psychiatric disorders, chronic disorders and obstetric complications were excluded from the study. The appropriate sample size for cross-sectional studies was calculated assuming a 99% confidence level (Z 1 − α⁄2 = 2.58), a presumed prevalence of compliance with IFA supplementation of 50% and a 5% margin of error. The minimum required sample size was 666. By adding a 10% non-response rate, a sample size of 733 was needed. Thus, 733 women were included in the study. Participants were selected by choosing every second name in the postnatal ward admission book (starting from 12 a.m. each day of data collection), until the required sample size was achieved. If the selected patient met the exclusion criteria, the subsequent name was selected instead.

### 2.2. Procedure

Data were obtained from the responses to an interviewer-administered questionnaire (Questionnaire S1). The questionnaire consisted of four parts; part 1: maternal socio-demographic data (maternal ethnicity, education level and family income), part 2: current and previous obstetric data, part 3: questions to assess maternal compliance to IFA supplementation and the reasons for noncompliance where necessary (side effects, family influences to not to take supplements, forgetfulness, negligence, thinking of no need to take the supplements after the symptoms relieved, fear that too much tablets will harm the health of herself or the baby’s, discoloration of teeth etc.) and part 4: neonatal data. The questions in part 3 were focused on the dietary compliance (main meals, consumption of tea and coffee), dosage, time, frequency, duration and practices of storage of IFA supplementation as well as the experience of side effects (gastrointestinal disturbances, vomiting, constipation and lack of appetite). Maternal compliance with antenatal iron, folic acid and calcium supplements, as well as maternal adherence to dietary advice, were also assessed during the interviews. In addition to the structured questionnaire, data were collected from the pregnancy charts and bedside notes of each participant and were used in data confirmation. Information regarding parity, history of an LBW infant, miscarriages and/or abortions, antenatal clinic visits, anthelminthic treatment and maternal haemoglobin levels during the current pregnancy was directly obtained from the pregnancy chart of each mother. A study pre-test was performed to check the validity, reliability and appropriateness of the questions and responses of the questionnaire. This was performed in September 2017 using a convenience sample of 10 postpartum women from a postnatal ward of the same institution. The questionnaire was revised and finalised after the provided data had been analysed and the 10 participants were excluded from the final cohort.

### 2.3. Data Management

The anaemia was classified in accordance with the WHO guidelines of haemoglobin levels, using the diagnosis anaemia at sea level during pregnancy. Accordingly, non-anaemia was defined as having a haemoglobin level ≥ 11.0 g/dL, while anaemia was defined as having haemoglobin level of <11.0 g/dL [5]. Compliance with antenatal IFA supplementation in this study was defined as daily intake of IFA supplements from the beginning of the second trimester until the day of hospital admission for impending delivery. This information was verified by asking the mothers whether they have taken the IFA supplements daily or not. The mothers who responded as ‘yes’ were considered as compliance with the IFA supplementation throughout the recommended period. As the recommended period is somewhat long (from the beginning of the 13th week of gestation until the hospital admission i.e., about 27 weeks), they were not questioned about the exact number of dates that they had taken the supplements within the time duration. Similarly, the mothers who had responded as ‘no’ for the above question were considered as non-compliance with the IFA supplementation with regard to the recommended period. In addition to irregularities of supplement consumption, non-compliance with the recommended supplementation was recorded when the woman had taken iron supplements together with calcium supplements and/or had not taken vitamin C together with the iron tablet. Non-compliance with dietary advice during the period of IFA supplementation was recorded when the woman had consumed tea, coffee and/or milk within one hour before or after iron supplement intake and/or main meals within one hour before or two hours after iron supplement intake.

### 2.4. Data Analysis

All analyses were performed using Minitab 19 statistical software. Descriptive statistics are presented as the mean ± standard deviation or as frequencies and percentages. All continuous variables were first assessed using numerical and graphical techniques (e.g., scatter plots) to confirm that they met the distributional assumption of the statistical tests used in their analysis. Univariate and multivariate binary logistic regression analyses were used to identify factors associated with maternal compliance with IFA supplementation during pregnancy. Univariate and multivariate analyses were carried out by regarding maternal compliance with IFA supplementation as the dependent variable; maternal age, education level, occupation, area of residence, family income, parity, history of an LBW infant, history of abortion, history of preterm delivery, history of anaemia and/or anaemia at the first antenatal clinic visit (initial visit) were regarded as independent variables to identify the factors affecting maternal compliance. First, the univariate analyses were used to assess the relationships between the proposed independent variables and IFA supplement compliance. Then the variables with a *p* value ≤ 0.1 in the univariate analyses were selected (maternal employment, maternal educational level, monthly family income, history of an LBW infant, history of abortion and history of anaemia) and assessed for the multicollinearity by running a linear regression analysis considering one of the selected independent variable as a dependent variable and remaining as independent variables. Variance inflation factor (VIF) was used to decide the multicollinearity and the variables with VIF < 5 were then included in multivariate logistic regression to identify factors independently associated with maternal IFA supplement compliance. The odds ratio (OR) and corresponding 95% confidence interval (CI) were calculated to assess the statistical significance of the associated variables. A *p* value ≤ 0.05 was considered to indicate statistical significance.

### 2.5. Ethics

Ethical approval for this study was obtained from the Institutional Ethics Review Committee, Provincial General Hospital Kurunegala, Sri Lanka (THK/HIRU/ERC/17/21). Permission to conduct the study was obtained from the Director of the Provincial GeneralHospital, Kurunegala, and the Consultant Obstetricians of the respective postnatal wards. The study was conducted in compliance with the principles of the Declaration of Helsinki. Informed written consent was obtained from all participants prior to their enrolment in the study.

## 3. Results

### 3.1. Characteristics of the Study Participants

Of the 733 postpartum women initially included in the study, 30 were excluded: 16 due to incomplete questionnaires and 14 due to missing third trimester haemoglobin test results. Therefore, the final cohort consisted of 703 women. Their mean age was 30.2 ± 5.1 years (range: 16 to 52 years). The educational level of half (50.5%) of the participants was the ordinary level. The monthly income of the majority of the participants exceeded 32,000 Sri Lankan rupees. The mean gestational age of the women at their initial visit to the ANC was 8.7 ± 2.7 weeks (Table 1).

### 3.2. Prevalence of Maternal Anemia

The mean blood haemoglobin concentrations were 11.6 ± 1.0 g/dL at the initial visit (gestational age, 8.7 ± 2.7) and 11.1 ± 0.9 g/dL at the third trimester (gestational age, 28.4 ± 2.3 weeks). Anaemia was diagnosed in 20.8% of the mothers at the first antenatal visit and in 44.9% at the third trimester. Compliance with all of the supplements, including iron, folic acid, vitamin C and calcium lactate, was observed in 84.2% of the participants throughout the required period, whereas 5.8% of the participants were non-compliant only with the recommended duration. Also iron supplements were taken together with calcium supplements by 3.8% of the women. Based on a definition of compliance with IFA supplementation as the daily intake of the IFA supplements together with vitamin C tablets at a time distinct from calcium supplementation and extending from the beginning of the second trimester until the day of hospital admission for impending delivery, the rate of maternal compliance with IFA supplementation was 80.1%.

### 3.3. Maternal Compliance with Dietary Advice

Analysis of compliance regarding dietary advice on tea, coffee and milk consumption, as well as main meal consumption, showed that only 26.6% of the participants adhered to the dietary recommendations while taking IFA supplements (Table 2).

### 3.4. Factors Associated with Compliance and Non-Compliance with IFA Supplementation

Forgetfulness (57.9%) was the major reason for non-compliance, followed by side effects (13.6%) and negligence (7.1%).

The most common side effect was nausea and vomiting (12.4%), with a frequency of 25% in the non-compliance group. Other side effects reported by the participants included constipation (12.1%), lack of appetite (3.1%) and gastrointestinal disturbance (0.6%). The results of multivariate analysis showed that women who were employed [OR: 1.7 (CI: 1.00–2.89)], had a history of an LBW infant [OR: 0.41 (CI: 0.19–0.90)] and had a history of anaemia [OR: 0.35 (CI: 0.12–0.98)] were more likely to be non-compliant with the recommended IFA supplementation during pregnancy (Table 3). The goodness of fit tests (Pearson and Hosmer-Lemeshow) for the fitted binary logistic regression model were greater than the significance level of 0.05, which indicates the model adequately fits the data.

Nearly all women (97.1%) were informed about the benefits of supplement usage during pregnancy, by means of group awareness programs held at ANCs. Furthermore, nearly all (96.3%) of the women had received the information through antenatal classes held at ANCs. However, only 17.8% of the participants had received instructions regarding the correct use of the supplements each time they received supplements from the clinic.

## 4. Discussion

Prevalence of anaemia during pregnancy has fluctuated around 30%in Sri Lanka in recent years, and remains as an important public health issue [3]. In Sri Lanka, 99.9% of deliveries are institutional deliveries [15] while 91.7% of them occur in government health institutions [16]. Because our study was conducted in a large tertiary care hospital in Sri Lanka, the cohort can be considered representative of women of child-bearing age in Sri Lanka, including considerations of income and area of residence. Women at postpartum days 0–4 were included because this allowed the collection of data regarding maternal compliance while avoiding recall bias. IFA supplementation programmes have been highly effective in reducing maternal anaemia worldwide [17,18]. Among our study population, maternal anaemia rates in the first and third trimesters were 20.8% and 44.9%, respectively. Because the first-trimester tests were performed at the very first ANC visit, their results reflect pre-pregnancy anaemia or a non-anaemic status.

However, the prevalence of third trimester anaemia was much higher in the present study than in the national statistics for year 2016 (35.36%) [3], although those country-specific statistics did not specify either gestational age or haemoglobin levels.

In the present study, 80.1% of the women were compliant with the recommended IFA supplementation during pregnancy; this rate was much higher than the rates reported from South Ethiopia [19,20,21], Kenya [22], Indonesia [23] and Iran [24]. This might be due to the comprehensive national maternal healthcare system in Sri Lanka, which targets all aspects of maternal and child health (e.g., maternal nutrition), and the provision of IFA supplements to all pregnant women throughout pregnancy under the totally free national healthcare services available in the country.

We also found that maternal non-compliance was 1.7-fold greater among employed women than among non-employed women [OR: 1.7 (CI: 1.00–2.89)]. There was no association between maternal age and IFA supplement compliance, consistent with the findings of studies in India [25] and some parts of Ethiopia [19,20,21], but not with the findings of a study from western Iran [24]. Other factors related to maternal non-compliance with IFA supplementation were a history of a LBW infant and a history of anaemia.

The major reason for non-compliance was forgetfulness, followed by side effects. Forgetfulness might be addressed by introducing strategies such as taking the supplements at a consistent time each day and placing them in a location that is readily accessible [19]. Policymakers may take the advantage of technology to overcome the forgetfulness by setting up electronic reminders such as mobile phone text alerts every day to remind them the time of taking supplementation. Forgetfulness was also cited as the reason for non-compliance in studies from Ethiopia [19], India [26] and Thailand [27]. Among the group of non-compliant women, side effects of nausea and vomiting were the most common reasons for not taking the IFA supplements. Some studies suggest that counselling regarding the importance of IFA supplementation and the frequency of ANC visits correlate positively with maternal compliance. In our study, nearly all of the mothers reported that they had received counselling regarding IFA supplements by their ANC; moreover, nearly all visited their ANC regularly.

Despite the high rate of maternal compliance with IFA supplementation in this study, the high prevalence of anaemia during the third trimester raises concern. This high prevalence of anaemia may reflect non-compliance with dietary advice during IFA supplementation. Sri Lanka is a country where tea drinking is a strong tradition, such that >85% of people regularly drink black tea [28]. Tea and coffee contain caffeine, which can interfere with iron absorption and should therefore be consumed at a separate time from IFA supplements, similar to the separate time for main meals. In addition, calcium can interfere with iron absorption; milk and other dairy products should thus be consumed at a separate time from IFA supplements. Specifically, for efficient iron absorption, IFA supplements should be taken on an empty stomach, preferably one hour before or two hours after the main meal; tea, coffee, milk and other dairy products should be consumed one hour before or after IFA supplement consumption. However, the women in our study had high rates of non-compliance with these dietary recommendations, which could explain the high prevalence of maternal anaemia during the third trimester of pregnancy. Dietary non-compliance would lead to poor efficacy of the IFA supplements, despite good compliance with their use. These shortcomings can be effectively addressed by ANCs, such as by educating pregnant women regarding the importance of compliance with IFA supplements and corresponding dietary recommendations. While nearly all mothers reported receipt of counselling regarding IFA supplementation through ANC educational classes, the content of antenatal educational classes should be revised to emphasise the importance of anaemia prevention, compliance with IFA supplementation and dietary compliance while taking IFA supplements.

### Limitations

A notable limitation of this study was its use of self-reported data regarding maternal compliance with IFA supplementation, which might have led to the underestimation or overestimation of compliance. Furthermore, the prevalence of maternal anaemia was determined based on secondary data available in the women’s pregnancy charts, which may have included reporting errors. Finally, there may have been inter-laboratory variations, because the blood tests were performed by multiple laboratories. It is therefore not possible to confidently provide inferences and recommendations based on only one cross-sectional study, so causation should always be confirmed by further studies, preferably of prospective design when possible.

## 5. Conclusions

The rate of maternal compliance with IFA supplementation was 80.1%. Maternal employment, history of an LBW infant and history of anaemia were significantly associated with maternal compliance. Nearly all of the pregnant women in our study had received IFA supplements, as well as free counselling regarding the use of those supplements, through community ANCs. This clearly demonstrates the dedication of Sri Lanka’s national maternal healthcare system to prevention and control of maternal anaemia. Nonetheless, the poor dietary compliance of the women may explain the high prevalence of maternal anaemia during the third trimester of pregnancy, despite the high rate of IFA supplementation compliance. Policymakers should revise the contents of the antenatal education program to strengthen the national programme for maternal anaemia prevention. Individualised education should be provided to women with symptoms of anaemia during their initial visit to the ANC.

## Figures and Tables

**Table 1:** Characteristics of participants (*n* = 703).

Variable		Mean (SD)	*n* (%)
Age (years)		30.2 (5.1)	-
Maternal Level of education ^a^			
	No school education or up to primary education	-	14 (2.0)
	Up to ordinary level	-	355 (50.5)
	Up to advanced level	-	269 (38.3)
	Higher education	-	65 (9.2)
Area of residence			
	Urban	-	153 (21.8)
	Sub-urban and villages	-	547 (77.8)
	Estate	-	03 (0.4)
Maternal employability			
	Not employed	-	587 (83.5)
	Employed	-	116 (16.5)
Family Income (LKR)			
	<14,000	-	26 (3.7)
	14,000–19,999	-	55 (7.9)
	20,000–31,999	-	190 (27.1)
	≥32,000	-	429 (61.3)
Family type			
	Nuclear family	-	444 (63.2)
	Extended family	-	258 (36.7)
Gestational age at first ANC visit (weeks)		8.7 (2.7)	-
Pre-pregnancy BMI			
	Underweight (BMI < 18.5 kg/m^2^)	-	122 (17.3)
	Normal (BMI 18.5 to 24.9 kg/m^2^)	-	392 (55.8)
	Overweight (BMI 25 to 29.9 kg/m^2^)	-	153 (21.8)
	Obese (BMI ≥ 30 kg/m^2^)	-	36 (5.1)
Parity			-
	Primiparous	-	224 (31.9)
	Multiparous	-	479 (68.1)
History of low birth weight deliveries			
	Yes	-	33 (4.8)
	No	-	659 (95.2)
History of pre-term deliveries			
	Yes	-	32 (4.6)
	No	-	658 (95.4)
History of abortions			
	Yes	-	122 (17.3)
	No	-	581 (82.7)
History of anaemia			
	Yes	-	17 (2.5)
	No	-	674 (97.5)
Number of total clinic visits		6.8 (2.0)	-
Distance to antenatal clinic (km)		2.6 (1.8)	-
Blood haemoglobin concentration at first ANC visit (g/dL)		11.6 (1.0)	-
Blood haemoglobin concentration at third trimester (g/dL) ^b^		11.1 (0.9)	-
Counselling on IFA supplements			
	Yes		604 (85.9)
	No		99 (14.1)
Anthelminthic treatment during pregnancy			
	Yes		695 (98.9)
	No		8 (1.1)

**^a^** The general education system in Sri Lanka provides 13 years school education; primary school (grade 1 to 5; age 6 to 10 years), ordinary level (grade 10 to 11; age 16 to 17 years), advanced level (grade 12 to 13; age 18 to 19 years), ^b^ mean gestational age at third trimester blood haemoglobin test was 28.4 ± 2.3 weeks. IFA: iron and folic acid; SD: standard deviation; LKR: Sri Lankan rupee; BMI: body mass index.

**Table 2:** Maternal compliance with dietary advices vs. compliance with IFA supplements and anaemic status.

		Compliance with Dietary Advices	
		Compliant *n* (%)	Non-Compliant *n* (%)
		187 (26.6)	516 (73.4)
Compliance with recommendations of IFA supplements	Compliant	146 (25.9)	417 (74.1)
	Non-compliant	41 (29.3)	99 (70.7)
Status of anaemia at first ANC visit	Anaemic (Hb < 11.0 g/dL)	39 (26.7)	107 (73.3)
	Not anaemic (Hb ≥ 11.0 g/dL)	148 (26.6)	409 (73.4)
Status of anaemia at third trimester	Anaemic (Hb < 11.0 g/dL)	92 (29.1)	224 (70.9)
	Not anaemic (Hb ≥ 11.0 g/dL)	95 (24.6)	292 (75.4)

IFA: iron and folic acid; Hb: haemoglobin.

**Table 3:** Multivariate analysis for maternal compliance with recommended IFA supplementation.

Variable in Model		Number of Subjects (*n* = 687)		Odds Ratio	95% CI	*p* Value
		Compliant with IFA (*n* = 548)	Non-Compliant with IFA (*n* = 139)			
Maternal age				0.96	0.93–1.00	0.073
Maternal employability						
	Not employed–reference level	464	109			
	Employed	84	30	1.7	1.00–2.89	0.048 *
Level of education						
	No school education or up to primary level– reference level	08	05			
	Up to ordinary level	279	68	0.53	0.14–1.92	0.331
	Up to advanced level	219	45	0.50	0.13–1.90	0.310
	Higher education	42	21	1.18	0.29–4.83	0.823
Monthly family income						
	<14,000 LKR–reference level	18	08			
	14,000–19,999 LKR	34	19	1.73	0.59–5.04	0.314
	20,000–31,999 LKR	151	36	0.73	0.27–1.96	0.529
	≥32,000 LKR	345	76	0.56	0.22–1.47	0.242
Having history of low birth weight						
	Yes–reference level	20	13			
	No	528	126	0.41	0.19–0.90	0.021 *
History of abortion						
	Yes–reference level	88	32			
	No	460	107	0.70	0.43–1.16	0.171
History of anaemia						
	Yes–reference level	10	07			
	No	538	132	0.35	0.12–0.98	0.046 *

The final multivariate logistic model included as a dependent variable the compliance (no-response event/yes) and as independent variables presented in the table; 687 cases were used as 16 cases contained missing values. CI: confidence interval; LKR: Sri Lankan rupee. * *p* < 0.05.

## Supplementary Materials

The following is available online at https://www.mdpi.com/2072-6643/12/11/3266/s1, Questionnaire S1: Maternal Compliance to Recommended Iron and Folic Acid Supplementation in Pregnancy, Sri Lanka: A Hospital Based Cross-Sectional Study.
