# Correction: Pathirathna et al. Maternal Compliance to Recommended Iron and Folic Acid Supplementation in Pregnancy, Sri Lanka: A Hospital-Based Cross-Sectional Study. *Nutrients* 2020, *12*, 3266

**DOI:** 10.3390/nu16070983

**Published:** 2024-03-28

**Authors:** Malshani L. Pathirathna, Kuruppu M. S. Wimalasiri, Kayako Sekijima, Mieko Sadakata

**Affiliations:** 1 Department of Nursing, Faculty of Allied Health Sciences, University of Peradeniya, Peradeniya 20400, Sri Lanka; 2 Department of Food Science and Technology, Faculty of Agriculture, University of Peradeniya, Peradeniya 20400, Sri Lanka; swarnaw@pdn.ac.lk; 3 Department of Nursing, School of Health Sciences, Niigata University, 2-746 Asahimachi-dori, Chuo-ku, Niigata 951-8518, Japan; ksekijima@clg.niigata-u.ac.jp (K.S.); sadakata@clg.niigata-u.ac.jp (M.S.)

## Table Legend

In the original publication [1], there was a mistake in the legend for Table 3. In the footnote, we stated, “The final multivariate logistic model included as dependent variable, the compliance (no/yes) and, as independent variables presented in the table; 687 cases were used as 16 cases contained missing values. CI: confidence interval; LKR: Sri Lankan rupee. * *p* < 0.05”.

The correct footnote appears below.

“The final multivariate logistic model included as a dependent variable the compliance (no-response event/yes) and as independent variables presented in the table; 687 cases were used as 16 cases contained missing values. CI: confidence interval; LKR: Sri Lankan rupee. * *p* < 0.05”.

## Text Correction

There was an error in the original publication’s [1] results. Under the Section 3.4, we stated, “The results of multivariate analysis showed that women who were employed [OR: 1.7 (CI: 1.00–2.89)], had a history of an LBW infant [OR: 0.41 (CI: 0.19–0.90)] and had a history of anaemia [OR: 0.35 (CI: 0.12–0.98)] were more compliant with the recommended IFA supplementation during pregnancy (Table 3)”.

A correction has been made to the Results section, Section 3.4, paragraph 2:

“The results of multivariate analysis showed that women who were employed [OR: 1.7 (CI: 1.00–2.89)], had a history of an LBW infant [OR: 0.41 (CI: 0.19–0.90)] and had a history of anaemia [OR: 0.35 (CI: 0.12–0.98)] were more likely to be non-compliant with the recommended IFA supplementation during pregnancy (Table 3)”.

There was an error in the original publication’s [1] Discussion section.

Corrections have been made to the Discussion section, paragraph 4:

In the original publication, paragraph 4 of the Discussion, we stated, “We also found that maternal compliance was 1.7-fold greater among employed women than among non-employed women [OR: 1.7 (CI: 1.00–2.89)]”.

The revised version should be read as “We also found that maternal non-compliance was 1.7-fold greater among employed women than among non-employed women [OR: 1.7 (CI: 1.00–2.89)]”.

In the original paper, paragraph 4 of the Discussion, the statement “This may be due to better planning and scheduling, including when to take the supplements, by working women” has been removed from the revised paper.

In the original paper, on page 8, paragraph 4 of the Discussion section, we stated, “Other factors related to a high rate of maternal compliance with IFA supplementation were a history of an LBW infant and a history of anaemia”.

The revised version should be read as “Other factors related to maternal non-compliance with IFA supplementation were a history of a LBW infant and a history of anaemia”.

In the original paper, paragraph 4 of the Discussion section, the statement “This could reflect greater caution among pregnant women in both groups to ensure a good pregnancy outcome” has been deleted from the revised version.

The authors apologize for any inconvenience caused and state that the scientific conclusions are unaffected. This correction was approved by the Academic Editor. The original publication has also been updated.
