# Supplementary material for: Maternal Compliance to Recommended Iron and Folic Acid Supplementation in Pregnancy, Sri Lanka: A Hospital-Based Cross-Sectional Study
Source: Nutrients. 2020 Oct 25;12(11):3266. doi: 10.3390/nu12113266 (PMC7694027; doi:10.3390/nu12113266)
Supplement: Supplementary file 1 [file nutrients-12-03266-s001.pdf]

Questionnaire S1

Reference No: ....

Maternal Compliance to Recommended Iron and Folic Acid Supplementation in Pregnancy, Sri Lanka: A Hospital Based Cross-Sectional Study

Part I – Socio demographic data

1. Maternal age:……………………years
2. Ethnicity:
   I. Sinhala ☐
   II. Tamil ☐
   III. Muslim ☐
   IV. Other ☐
3. Level of maternal education:
   I. No school education ☐
   II. Up to primary level ☐
   III. Up to secondary level ☐
   IV. Higher ☐
4. Area of residence:
   I. Urban ☐
   II. Sub-urban ☐
   III. Rural ☐
5. Occupation:
   I. Homemaker ☐
   II. Working mother ☐ ; Type of work……………………………
6. Monthly household income:
   I. Less than 9000 LKR ☐
   II. 9000- 13999 LKR ☐
   III. 14000-19999 LKR ☐
   IV. 20000-31999 LKR ☐
   V. More than 32000 LKR ☐

7. Family type:
   I. Nuclear family type ☐
   II. Extended family type ☐

Part II – Obstetrics data

8. Pre-pregnancy BMI:……………………..kg/m^2
9. Body weight at first antenatal clinic visit:………………………..kg; at………………gestational weeks
10. Body weight at last antenatal clinic visit:………………………..kg; at………………gestational weeks
11. Total gestational weight gain: ……………………..kg
12. Parity:
    I. Primiparous ☐
    II. Multiparous ☐ ; If multiparous, birth order:……………………..
13. Birth interval/Pregnancy interval (if the previous pregnancy was a miscarriage or abortion):………………….
    (Please delete the irrelevant part using one line)

14. Regular antenatal clinic visit for this pregnancy:
    I. Yes ☐
    II. No ☐
15. Total number of antenatal clinic visit during this pregnancy:………………………..
16. How far from your house to antenatal clinic:……………………Km
17. Anti-helminth treatment during this pregnancy:
    I. Yes ☐ ; At gestational age………………..weeks
    II. No ☐
18. History of prophylaxis treatment for malaria
    I. Yes ☐ ; When……………………
    II. No ☐
19. Previous history of low birth weight delivery:
    I. Yes ☐ ; if yes, birth weight………………..g
    II. No ☐

20. Previous history of preterm delivery:

    I. Yes ☐ ; if yes, reason for preterm delivery……………………..; gestational age at delivery…….weeks

    II. No ☐

21. History of miscarriage or abortion:

    I. Yes ☐

    II. No ☐

22. History of anemia during previous pregnancies:

    I. Yes ☐

    II. No ☐

23. History of any intervention for treatment of anemia as injection Iron or blood transfusion in previous pregnancy?

    I. Yes ☐

    II. No ☐

24. History of any intervention for treatment of anemia as injection Iron or blood transfusion during this pregnancy?

    I. Yes ☐

    II. No ☐

25. Hemoglobin test results at booking visit (this pregnancy):……………..g/dl; at ………………..gestational weeks

26. Hemoglobin test results at third trimester (this pregnancy):……………..g/dl; at ………………..gestational weeks

Part III – Maternal compliance to antenatal iron, Folic acid and calcium supplementations

27. Did you receive the advices to add food items abundant with iron for your meals?

    I. Yes ☐

    II. No ☐

28. Did you explanations on what benefits you can have from each supplement (Iron, Folic acid, Vitamin C and Calcium Lactate)?

    I. Yes ☐

    II. No ☐

29. Did you receive the instructions (dose, time, frequency, how long, storage etc.) for taking each tablet?

    I. Yes ☐

    II. No ☐

30. Did you receive the explanation on possible side effects of iron tablet intake before or at the time of receiving tablets?

    I. Yes ☐
    II. No ☐

31. Did you receive the instructions on how to reduce side effects if they occurred?

    I. Yes ☐
    II. No ☐

32. Did you receive the instructions/explanation regarding micronutrient supplements on;

    I. Individual basis ☐
    II. Group/ common basis ☐

33. When did you receive the explanation/instructions regarding supplements?

    I. When I received the supplements for the first time during this pregnancy:
        a. Yes ☐
        b. No ☐
    II. Every time that I received supplements during this pregnancy:
        a. Yes ☐
        b. No ☐
    III. Along with my hemoglobin test results:
        a. Yes ☐ ; if yes at what gestational age……………………
        b. No ☐
    IV. During antenatal educational sessions?
        a. Yes ☐ ; if yes, at what educational session (First/ Second/Third)
        b. No ☐

34. Which of the following tablet/s have you taken during this pregnancy?

    I. Folic acid ☐
    II. Iron-folate /Iron ☐
    III. Vitamin C ☐
    IV. Calcium Lactate ☐

35. When did you start to take each of following supplement?

I. Folic acid
   a. Before becoming pregnant ☐ b. After the gestational weeks…………………

II. Iron-Folate/Iron
   a. Before becoming pregnant ☐ b. After the gestational weeks…………………

III. Vitamin C
   a. Before becoming pregnant ☐ b. After the gestational weeks…………………

IV. Calcium Lactate
   a. Before becoming pregnant ☐ b. After the gestational weeks…………………

36. Did you intake calcium tablet separately from other supplement tablets?

I. Yes ☐

II. No ☐

37. If yes, how long the time gap between calcium tablet intake and Iron-folate tablet intake?

I. Time gap is within recommended limit ☐ ; ……………………….hours

II. Time gap is not within recommended limit ☐ ; ……………………….hours

38. Did you take the supplements daily?

I. Yes ☐

II. No ☐

39. How long did you continue to take supplements?.......................................

40. What was your usual time gap between main meals and Iron-folate tablet intake?

I. Before main meals ☐………hrs

II. After main meals…..☐…………hrs

41. What was your usual time gap (before or after) between Iron-folate supplement intake and <u>tea</u> consumption?

I. Time gap is within recommended limit ☐ ;……………………hours

II. Time gap is not within recommended limit ☐ ;……………………hours

III. Tea consumption pattern:

   a. Frequency:……..times/day

   b. Amount:……………….ml at one time

42. What was your usual time gap (before or after) between Iron-folate supplement intake and <u>coffee</u> consumption?

I. Time gap is within recommended limit ☐ ;.......................hours

II. Time gap is not within recommended limit ☐ ;.......................hours

III. Cofee consumption pattern:

a. Frequency:........times/day

b. Amount:...................ml at one time

43. What was your usual time gap (before or after) between Iron-folate supplement intake and milk consumption?

I. Time gap is within recommended limit ☐ ;.......................hours

II. Time gap is not within recommended limit ☐ ;.......................hours

III. Milk consumption pattern:

a. Type of milk ....(Fresh milk/Powdered milk/ Malted milk)

b. Frequency:........times/day

c. Amount:...................ml at one time

44. Did you take any medications for long time while you are taking iron-folate supplements?

I. Yes ☐ ; if yes, what type of medication?.............................

II. No ☐

45. Did you store each of the tablets separately in glass bottles/ plastic containers and keep lid closed tightly to prevent air entering in to the container:

I. Yes ☐

II. No ☐

46. Did you use a dark colored bottle for storing iron/iron folate tablets?

I. Yes ☐

II. No ☐

47. From where did you receive the supplements?

I. From community antenatal clinic ☐

II. Buy from a pharmacy ☐

48. Did you receive enough tablets in each time until your next clinic visit?

I. Yes ☐

II. No ☐

49. If you stopped taking supplement tablets or if you were unable to take tablets as advised, what are the reasons for not taking supplements as recommended?

    I. Side effects ☐
    II. Family influence to not to take supplements ☐
    III. Forgetfulness ☐
    IV. Negligence ☐
    V. Thinking of "no need to take after the symptoms relieved" ☐
    VI. Fear that too many tablets would harm mother and/or baby ☐
    VII. Discoloration of the teeth ☐
    VIII. Other…………………………………………………………………………………… ☐

50. If you continued to take supplement tablets as advised, what are the motivational factors for taking supplements as recommended?

    I. I do not want any unwanted outcome for my baby ☐
    II. I wanted a healthy pregnancy ☐
    III. I strongly trust instructions given by my health care provider ☐
    IV. I wanted to relieve from the anemic symptoms ☐
    V. Other…………………………………………………………………………………………

51. Did you experience any of the following side-effects after taking Iron-folate supplements?

    I. Gastrointestinal discomfort ☐
    II. Nausea ☐
    III. Vomiting ☐
    IV. Constipation ☐
    V. Loss of appetite ☐

<u>Part IV</u> <u>– Neonatal data</u>

52. Gestational age:……………………weeks
53. Sex: Male ☐ Female ☐
54. Birth Weight:……………………………g
55. Length at birth………………………cm
56. OCP at birth…………………………..cm

57. Mode of delivery

I. Normal Vaginal Delivery ☐

II. Cesarean section Delivery ☐

a. Elective ☐

b. Emergency ☐

III. Forceps Assisted Delivery ☐

IV. Vacuum Delivery ☐

~~END~~
